# FABP4 in obesity-associated carcinogenesis: Novel insights into mechanisms and therapeutic implications

**DOI:** 10.3389/fmolb.2022.973955

**Published:** 2022-08-19

**Authors:** Shujie Liu, Dong Wu, Zhiyao Fan, Jian Yang, Yongzheng Li, Yufan Meng, Changhao Gao, Hanxiang Zhan

**Affiliations:** Division of Pancreatic Surgery, Department of General Surgery, Qilu Hospital, Shandong University, Jinan, China

**Keywords:** FABP4, fatty acids, obesity, cancer, macrophage

## Abstract

The increasing prevalence of obesity worldwide is associated with an increased risk of various diseases, including multiple metabolic diseases, cardiovascular diseases, and malignant tumors. Fatty acid binding proteins (FABPs) are members of the adipokine family of multifunctional proteins that are related to fatty acid metabolism and are divided into 12 types according to their tissue origin. FABP4 is mainly secreted by adipocytes and macrophages. Under obesity, the synthesis of FABP4 increases, and the FABP4 content is higher not only in tissues but also in the blood, which promotes the occurrence and development of various cancers. Here, we comprehensively investigated obesity epidemiology and the biological mechanisms associated with the functions of FABP4 that may explain this effect. In this review, we explore the molecular mechanisms by which FABP4 promotes carcinoma development and the interaction between fat and cancer cells in obese circumstances here. This review leads us to understand how FABP4 signaling is involved in obesity-associated tumors, which could increase the potential for advancing novel therapeutic strategies and molecular targets for the systematic treatment of malignant tumors.

## 1 Introduction

Obesity has become increasingly prevalent worldwide. Over the past few decades, the global prevalence of obesity and overweight has consistently increased by over 27% in adults (Avgerinos et al., 2019). In 2013, >35% of adults and >20% of children were overweight or obese, with the numbers being raised constantly (Quail and Dannenberg, 2019). Overweight was defined as a body mass index (BMI) ≥25 kg/m^2^ and <30 kg/m^2^, and obesity was defined as BMI ≥30 kg/m^2^ (Avgerinos et al., 2019; Piche et al., 2020). Obesity is known to cause a chronic low-grade systemic inflammatory response in the human body, which can lead to a series of diseases (Stolarczyk, 2017), most commonly referred to as hypertension, cardiovascular disease, dyslipidemia, metabolic syndrome, type 2 diabetes (T2D) mellitus, Alzheimer’s disease, non-alcoholic fatty liver disease, cancers, and other chronic diseases (Avgerinos et al., 2019). Several previous studies have revealed that the changes caused by obesity in the fat tissue microenvironment play a pivotal role in the development and progression of numerous malignancies (Zhou et al., 2019).

When lipid anabolism and catabolism maintain a balance, they meet the metabolic needs of the body. In obese people, lipid metabolism is disordered, and the lipid/liposome balance is interrupted, resulting in excessive activation of adipose production and excessive accumulation of lipids (Cheng et al., 2019). Obesity is the process of excessive accumulation of fat in the body. Lipid metabolism disorders are the main pathophysiological changes that are associated with obesity. The enlargement of adipose tissues is mainly caused by hypertrophy of adipocytes, as the number of adipocytes is basically stable in adults, although in some cases there is adipocyte hyperplasia, such as excessive calorie intake. Adipocyte hypertrophy promotes inflammatory response, increasing secretion of pro-inflammatory adipokines like tumor necrosis factor (TNF) and interleukin (IL)-6 and decreasing secretion of anti-inflammatory adipokines like adiponectin and leptin. However, creating new small adipocytes reduces hypoxic stress and thus inflammation. For the adipocytokine FABP4, it has been shown that lipid accumulation drives its expression (Ghaben and Scherer, 2019). Excessive accumulation of fat in the body increases the release of free fatty acids (FFAs) into the blood. Fatty acids (FAs) are esterified to form triglycerides and lead to hypertriglyceridemia, resulting in hypercholesterolemia, in which the levels of very low- and high-density lipoprotein cholesterol increase and decrease, respectively (Deng and Liu, 2003). Abnormal lipid metabolism can harm multiple organs, including the liver, heart, and pancreas, thereby increasing the risk of diabetes, cardiovascular disease, and carcinoma (Pavlic and Lewis, 2011; Yazici and Sezer, 2017). Adipose tissue secretes adipokines and cytokines, such as adiponectin, TNF-α, leptin, and IL-6, which regulate metabolism, energy intake, and fat storage (Stolarczyk, 2017). In addition, several novel adipokines have been identified, including FABP4, apelin, endotrophin, visfatin, lipocalin 2, osteopontin, ANGPTL2, omentin-1, and chemerin, the roles of which as mediators between obesity and cancer have also been reported (Cabia et al., 2016).

FABPs are a family of multifunctional proteins that are associated with FA metabolism. The FABPs family is classified and named according to their tissue of origin (Chmurzyñska, 2006; Hotamisligil and Bernlohr, 2015) (Table 1). In general, 12 different FABPs family members have already been identified, two of which are not expressed in humans (Amiri et al., 2018). FABPs have traditionally been small, structurally conserved, water-soluble cytoplasmic proteins, with a relative molecular mass of ∼15,000 (Xu et al., 2006). Their family members share a common tertiary structure despite their different protein sequences, in which the central hydrophobic core is encircled by 10 antiparallel chains in two vertical directions, forming a twisted barrel-like structure called the β-barrel (Xu et al., 1993). Differences in protein sequences determine the ability of individual members to bind ligands. FABP4, also known as adipocyte FABP (A-FABP), is mainly secreted by adipocytes and macrophages (Li et al., 2020). FABP4, a typical intracellular lipid chaperone, is responsible for promoting lipid storage, distribution, transportation, decomposition and metabolism (Li et al., 2020). However, excess fat cells in obese people lead to elevated levels of FABP4, which has adverse downstream effects on various tissue types, including pancreatic endocrine cells, cardiovascular cells, and the liver (Hotamisligil and Bernlohr, 2015; Zhang et al., 2017). Many previous studies have shown that FABP4 is relevant to the development of metabolic diseases (e.g., atherosclerosis, insulin resistance, T2D), cardiovascular disease, asthma, and cancer (Engl et al., 2008; Prentice et al., 2019; Li et al., 2020; Lee et al., 2021a). Based on the above, FABP4 is closely linked to malignancies, especially obesity-related malignancies; however, there are only few retrospective analyses and reviews in this area. Hence, we aimed to investigate the role of FABP4 in obesity-associated carcinogenesis in terms of its molecular mechanisms and therapeutic significance. Here, we review recent findings focused on the role of FABP4 in lipid metabolism, particularly lipid metabolism, in obesity, the possible molecular mechanism of carcinogenesis, and potential therapeutic targets, including breast, ovarian, liver, stomach, and pancreatic cancers.

**TABLE 1:** FABP family members and their functions.

	Common name	Site of expression	Functions	Role in disease
FABP1	Liver FABP (L-FABP)	Liver, intestine, pancreas (Chmurzyñska, 2006; Amiri et al., 2018)	Lipid metabolism, energy homeostasis (Chmurzyñska, 2006; Santos and Schulze, 2012)	Hepatic steatosis, cancers (Santos and Schulze, 2012; Li et al., 2020)
FABP2	Intestinal FABP (I-FABP)	Small intestine (Darimont et al., 2000; Bingold et al., 2015)	Dietary lipid absorption (Darimont et al., 2000; Li et al., 2020)	Metabolic syndromes, intestinal malignancy (Thumser et al., 2014; Bingold et al., 2015)
FABP3	Heart FABP (H-FABP)	Cardiac and skeletal muscle, brain (Wang et al., 2014; Amiri et al., 2018)	Muscle lipid metabolism, heart development (Chmurzyñska, 2006; Wang et al., 2014)	A biomarker for congestive heart failure and ischemic heart disease (Wang et al., 2014; Amiri et al., 2018; Li et al., 2020)
FABP4	Adipocyte FABP (A-FABP)	Adipocytes, macro-phages, monocytes, and endothelial cells (Hotamisligil and Bernlohr, 2015; Li et al., 2020; Lee et al., 2021a)	Lipid storage, lipolysis, and metabolism (Chmurzyñska, 2006; Hotamisligil and Bernlohr, 2015; Amiri et al., 2018; Lee et al., 2021a)	Insulin resistance, type 2 diabetes mellitus and cardiovascular disease, carcinoma (Chmurzyñska, 2006; Hotamisligil and Bernlohr, 2015; Amiri et al., 2018; Li et al., 2020; Lee et al., 2021a)
FABP5	Epidermal FABP (E-FABP)	Skin epidermis, fat cells, macrophages, and dendritic cells (Fang et al., 2010; Dallaglio et al., 2013; Amiri et al., 2018)	Adjusting cellular fatty-acid movement, and skin metabolism and blood circulation (Fang et al., 2010; Dallaglio et al., 2013)	Inflammatory skin diseases, cancer, atherosclerosis, autoimmune diseases (Fang et al., 2010; Dallaglio et al., 2013; Amiri et al., 2018; Li et al., 2020)
FABP6	Ileal FABP (IL-FABP)	Ileum (Hendrick et al., 2016)	Existed in ileal, as a transporter of bile acids (Hendrick et al., 2016; Amiri et al., 2018)	Related to Colorectal cancer, type 2 diabetes (Chmurzyñska, 2006; Hendrick et al., 2016; Amiri et al., 2018)
FABP7	Brain FABP (B-FABP)	Brain, glial cells (Chmurzyñska, 2006; Kaloshi et al., 2007)	Neurogenesis, astrocyte proliferation (Chmurzyñska, 2006; Kaloshi et al., 2007; Amiri et al., 2018)	Down’s syndrome, Schizophrenia, cancers (Kaloshi et al., 2007; Amiri et al., 2018)
FABP8	Myelin FABP (M-FABP)	Myelin, Scwann cells (Chmurzyñska, 2006; Thumser et al., 2014; Amiri et al., 2018)	Regulates the structural and functional integrity of myelin (Chmurzyñska, 2006; Amiri et al., 2018; Li et al., 2020)	Guillain-Barre syndrome (Chmurzyñska, 2006; Thumser et al., 2014; Amiri et al., 2018; Li et al., 2020)
FABP9	Testis FABP (T-FABP)	Testis, salivary gland (Al Fayi et al., 2016)	Spermatogenesis and fertilization (Chmurzyñska, 2006; Al Fayi et al., 2016; Amiri et al., 2018)	Prostate cancer (Al Fayi et al., 2016; Amiri et al., 2018; Li et al., 2020)

## 2 FABP4 expression and functions

FABP4 exhibits some common characteristics with other members of the FABPs family but also have its own characteristics. Using fluorescence *in situ* hybridization and somatic cell hybridization, the human *FABP4* gene was confirmed to be located on chromosome 8q21 (Prinsen et al., 1997). FABP4 has a tertiary structure with family commonality which a hydrophobic core is surrounded by a twisted barrel composed of 10 antiparallel-strands. Comparing the amino acid sequences of FABPs, their binding ability differs according to different clustering. The binding ability of FABP4 is between FABP1 and FABP2, which could be combined with FAs and additionally eicosanoids and retinoids (Chmurzyñska, 2006). FABP4 has been confirmed to be expressed mainly in differentiated adipocytes and macrophages (Amiri et al., 2018), which are distributed in organs and tissues, such as adipose tissue, ciliary ganglion, appendix, skin, fetal thyroid, and placenta (Chmurzyñska, 2006). Recently published studies have also confirmed FABP4 expression in cancer cells, like breast cancer, ovarian cancer, colon cancer and so on (Maruthur et al., 2012; Mukherjee et al., 2020; Tian et al., 2020; Zeng et al., 2020). Notably, adipocytes primarily express FABP4, whereas FABP5 expression in adipocytes is increased when FABP4 is deficient (Li et al., 2020). In addition, FABP4 is expressed in macrophages, mediating the inflammatory response and cholesterol accumulation (Makowski et al., 2001), while FABP5 does not show compensatory compensation (Li et al., 2020).

FABP4, also named adpocyte protein 2, which are abundantly expressed in fat cells, maintain adipocyte homeostasis and regulate adipocyte metabolism by interacting with peroxisome proliferator-activated receptor-γ (PPAR-γ) and hormone-sensitive lipase (HSL) (Prentice et al., 2019). FABP4 has a HSL binding site (Smith et al., 2008), through which can interact with each other. It mainly acts as a transporter protein in the cell (Schwenk et al., 2010) by binding to FAs that enter the plasma membrane to facilitate their intracellular transport and regulate glucose and lipid metabolism by upregulating FAs. The main function of FABP4 is to regulate the metabolism of FAs, which can act as signaling molecules to regulate FABP4 expression. FABP4 increases FA concentration and promotes the mRNA expression of related transcription factors encoding FABP4 (Distel et al., 1992; Cabia et al., 2016). Multiple studies have shown that FABP4 expression is influenced by FAs, agonists, or inhibitors of PPAR-γ, and insulin by controlling the transcription of its differentiation-related mRNA (Jenkins-Kruchten et al., 2003; Haunerland and Spener, 2004; Pelsers et al., 2005; Furuhashi and Hotamisligil, 2008; Schroeder et al., 2008; Robinson et al., 2009).

**FIGURE 1:**
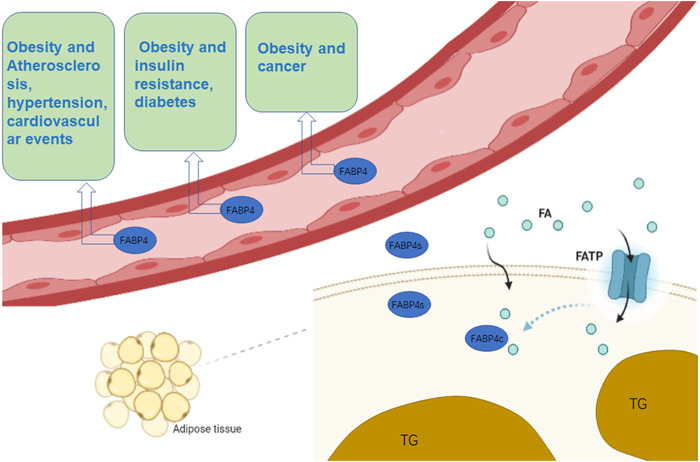
The role of FABP4 in cells and blood. n adipocytes, FABP4 binds to FAs that enter the cell membrane and assist in their transport. Simultaneously, fat cells synthesize FABP4 and release part of it into the blood. Circulating FABP4 plays an undeniable role on atherosclerosis, hypertension, cardiovascular events, insulin resistance, diabetes and cancer. (Some of the elements in the figure come from BioRender.)

Although FABP4 has traditionally been thought of as a cytoplasmic protein, several studies have demonstrated that it can be released into the bloodstream (Xu et al., 2006) to act as a specific circulating FABP4. Plasma FABP4 levels are significantly elevated in obese individuals (Cao et al., 2013), possibly because of increased secretion per fat mass. In addition, potential changes in tissue absorption and clearance due to inflammation also contribute to elevated plasma FABP4 levels (Cao et al., 2013). Exalted circulating FABP4 content is strongly related to obesity, atherosclerosis, cardiovascular events, insulin resistance, hypertension and diabetes (Xu et al., 2006; Xu et al., 2007; Furuhashi et al., 2014; Hao et al., 2018a; Prentice et al., 2019; Lee et al., 2021a) (Figure 1). Other members of the FABPs family have also been found circulating in the bloodstream and can be used as biomarkers for related tissue damage (Tanaka et al., 1991; Setsuta et al., 2002; Pelsers et al., 2003; Pelsers et al., 2004; Pelsers et al., 2005).

The role of FABP4 in cardiovascular and metabolic diseases has been widely investigated and some achievements have been made. It has been reported that FABP4 can be used as an effective clinical biomarker (Furuhashi et al., 2014) and that FABP4 inhibitors have broad therapeutic prospects (Floresta et al., 2017). A genome-wide association study of more than half a million people worldwide confirmed that FABP4 is a common risk factor for coronary heart disease and T2D (Zhao et al., 2017). Additionally, a basic experiment illustrated that FABP4 deficiency has a significant protective effect on atherosclerosis (Erbay et al., 2009). Mice lacking the *Fabp4* gene have a lower chance of developing atherosclerosis (Erbay et al., 2009). To date, there are none identified cases that lack *FABP4* gene expression; however, the health of FABP4 knockout mice is not affected (Prentice et al., 2019). Mechanistically, IL-6 and IL-10 exert anti-atherosclerotic effects (Kleemann et al., 2008; Madan et al., 2008). A previous study showed that in FABP4 knockout mice, the IL-6 and IL-10 signaling pathways were enhanced and unchanged, respectively. The same study also showed that binding of FABP4 with ligand FAs can interact with unphosphorylated Janus kinase 2 (JAK2) to attenuate JAK2 signaling, a process that requires the involvement of FAs (Thompson et al., 2009). Therefore, it can be speculated that FABP4 binding with FAs inhibits JAK2 signaling and decreases IL-6 levels, thus limiting the anti-atherosclerosis function and contributing to the occurrence of cardiovascular diseases. In 1996, a study revealed that FFAs are relevant to the induction of insulin resistance and FABP4 plays a pivotal role in the connection between obesity and insulin resistance (Cressman et al., 1996). It has been confirmed that FABP4-deficient mice produces less insulin (Furuhashi, 2019) and shows reduced insulin resistance (Uysal et al., 2000). Lipid analysis showed an increase in short-chain FAs but a decrease in long-chain FAs in mice with deficiency of FABP4 (FABP4^-^ mice) (Furuhashi and Hotamisligil, 2008). This FAs change facilitates the enhancement of insulin receptor signal transduction, insulin-stimulating glucose uptake, and promotes adenosine monophosphate-activated protein kinase activity and oxidation of FAs (Maeda et al., 2005). Therefore, we can also speculate that insulin resistance occurs when FABP4 levels are high. In addition to its effect on insulin, one study found that circulating FABP4 directly regulates glucose synthesis and metabolism in liver cells by increasing the expression of two gluconeogenic enzymes, glucose-6-phos-phatase and phosphoenolpyruvate carboxykinase 1. Therefore, the researchers pointed that FABP4 inhibitor therapy may be a beneficial strategy for the treatment of diabetes (Cao et al., 2013). A subsequent study confirmed this hypothesis, demonstrating that FABP4 small-molecule inhibitors were effective in treating severe atherosclerosis and T2D in mouse experimental models (Furuhashi et al., 2007), suggesting that chemical inhibition of FABP4 could also be utilized to produce beneficial effects in human diabetes and cardiovascular disease.

## 3 FABP4 in obesity associated carcinogenesis

**FIGURE 2:**
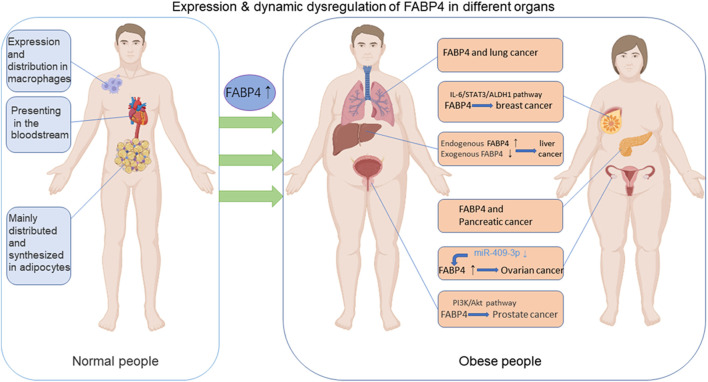
The functions of FABP4 in normal and obese people. FABP4 is synthesized and distributed mainly in adipocytes and macrophages and could be secreted into the blood circulation. FABP4 levels are elevated in obese individuals in comparison with normal people. FABP4 levels have been associated with carcinogenesis, including breast, ovarian, prostate, liver, pancreatic, lung cancers and so on through various pathways. (Some of the elements in the figure come from BioRender.).

Excessive accumulation of fat causes obesity, which has detrimental effects on human health (Piche et al., 2020). Cancer is a collection of diseases in which normal cellular metabolism undergoes uncontrollable neoplastic transformation through a string of incremental processes that arise under certain pressures (Chabner and Roberts, 2005). Owing to the sharp increase in the incidence of obesity, the association between obesity and cancer has recently received a lot of attention (Park et al., 2014; Zhou et al., 2019; Lega and Lipscombe, 2020; Ringel et al., 2020). Obesity is a common risk factor for several types of cancer, including ovarian (Mukherjee et al., 2020), breast (Brown, 2021; Lohmann and Goodwin, 2021), liver (Marengo et al., 2016), endometrial (Onstad et al., 2016), pancreatic (Paternoster and Falasca, 2020), colon (Frezza et al., 2006), renal (Capitanio et al., 2019) and prostate (Buschemeyer and Freedland, 2007) cancers (Figure 2). Although there is increasing evidence for the association between cancer and obesity, the underlying mechanisms remain contentious (Deng et al., 2016).

In fact, various classical adipokines, including adiponectin, leptin, TNF-α, IL-6, and resistin, which are significantly increased in obese patients, have been demonstrated to play pro-tumorigenic roles (Hefetz-Sela and Scherer, 2013; Ackerman et al., 2017; Cha and Koo, 2018; He et al., 2018; Quail and Dannenberg, 2019). Adiponectin and leptin are the most studied adipokines. Individuals with obesity exhibit higher leptin and lower adiponectin levels. A more aggressive malignant phenotype is associated with a higher leptin/adiponectin ratio (Grossmann et al., 2010). Additionally, adipokine lipocalin-2 regulates insulin resistance and adipose tissue inflammation (Yan et al., 2007). Besides these factors, additional adipokines have recently been identified and characterized using new proteomic techniques and approaches (Spyrou et al., 2018; Estienne et al., 2019) (Table 2). Altered FABP expression has been suggested as a potential mediator of carcinogenesis (McKillop et al., 2019; Jin et al., 2021). There is increasing evidence that FABPs act as promoters of cancer (McKillop et al., 2019). Guaita-Esteruelas et al. (2018) elucidated the role of FABP4 in the tumor microenvironment (TME) to promote carcinogenesis and the development of carcinoma. FABP4 has been proven to promote breast (Li et al., 2019), prostate (Liu and Godbout, 2020), ovarian (Nieman et al., 2011) cancers and other malignant tumors (Figure 2). Several signaling pathways regulated by FABP4 are involved in the obesity/cancer axis through complex cellular and molecular mechanisms. The following section focuses on the emerging roles of FABP4 during initial cell transformation and consecutive tumor progression.

**TABLE 2:** Novel adipokines that may be involved in promoting cancer.

Cytokines	Production site	Functions
Apelin	Adipocytes	Angiogenic factor (Picault et al., 2014; Cabia et al., 2016)
Endotrophin	Extracelullar matrix	Enhance mitotic activity (Park and Scherer, 2012; Spyrou et al., 2018)
Visfatin	Macrophages in adipose tissue	Pro-inflammatory role (He et al., 2012)
Lipocalin 2	Adipocytes	Favouring activity of MMP-9 and promoting cancer progression (Zhang et al., 2008; Cabia et al., 2016)
FABP4	Adipocytes and macrophages	Tumor cells ingest fatty acids (Xu et al., 2006; Wu et al., 2014)
Osteopontin	Macrophages in adipose tissue	Inducing the activation of matrix metalloproteinase-9 and matrix metalloproteinase-2 (Rangaswami et al., 2006; Cabia et al., 2016)
ANGPTL2	Adipocytes	Pro-inflammatory role (Aoi et al., 2011; Estienne et al., 2019)
Omentin-1	Macrophages in adipose tissue	Anti-inflammatory effects (Zhang and Zhou, 2013)
Chemerin	Adipocytes	Pro-inflammatory role (Kaur et al., 2010; Cabia et al., 2016)

**FIGURE 3:**
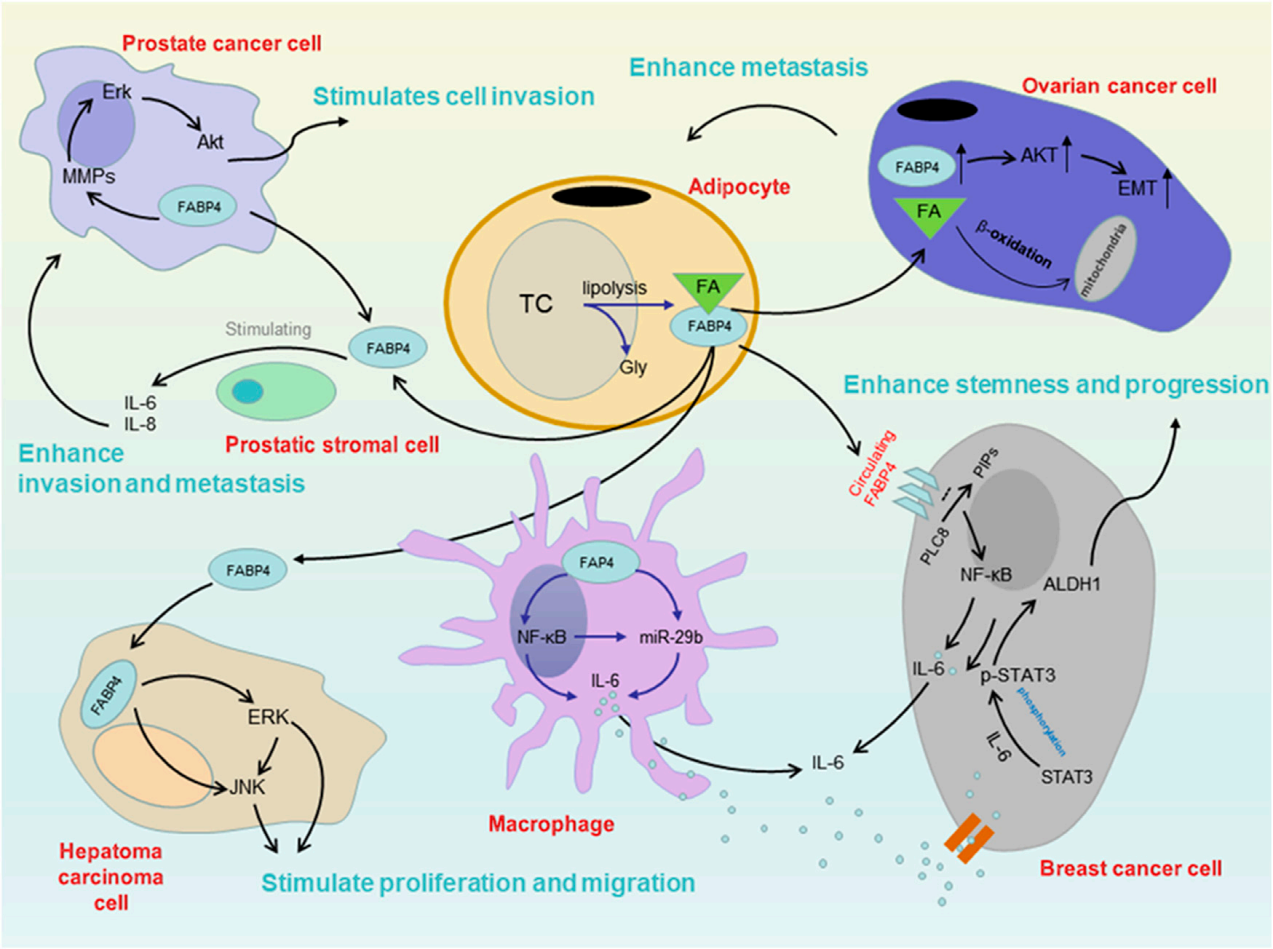
Mechanism between FABP4 and several cancers in obesity. There are some findings about the mechanism. Circulating FABP4 functions as a tumor promoting factor obesity-associated breast cancer through the IL-6/STAT3/ALDH1 pathway. FABP4 could additionally activate the PI3K/AKT pathway participating in the tumor growth and survival, such as prostate cancer. The rise of FABP4 leads to a series of metabolic changes in cells, resulting in a significant reduction of miR-409-3p expression, which promotes the occurrence and development of ovarian cancer. Moreover, FABP4 could stimulate the proliferation and migration of HCC cells, promoting liver cancer progression.

FABP4 is traditionally considered a cytoplasmic protein that coordinates lipid metabolism inside cells (Hotamisligil and Bernlohr, 2015). Recent studies have demonstrated that adipocytes and macrophages can secrete FABP4 when induced by external factors, and circulating FABP4 could function as a novel adipokine linking obesity-associated diseases (Hao et al., 2018a; Li et al., 2019). Thus, dysregulation of intracellular and extracellular/circulating FABP4 expression could be critical to tipping the scales towards the development of malignancy, both locally and systemically (Li et al., 2020) (Figure 3).

### 3.1 Breast cancer

Various risk factors, including inherited background, aging, reproductive history, and obesity are involved in the development and progression of breast cancer. Obesity predicts a worse prognosis for women of all ages and remarkably, a higher risk of breast cancer (Grossmann et al., 2010; Picon-Ruiz et al., 2017; Zeng et al., 2020).

FABP4 regulates inflammatory responses by triggering c-Jun N-terminal kinase and nuclear factor kappa B (NF-κB) pathways in macrophages. In contrast, FABP4 interacts with HSL and PPARγ in adipocytes to promote lipolysis and inhibit lipogenesis. Results from both mouse models and clinical studies support that FABP4 plays a key role in promoting obesity-related breast cancer development (Zeng et al., 2020). FABP4 protein expression was remarkably higher in obese adipose tissue than in lean controls. Additionally, obese patients with breast cancer displayed significantly higher FABP4 levels in serum, which were associated with larger tumor size and poorer prognosis, regardless of menopausal status (Hao et al., 2018a; Hao et al., 2018b). Several molecular and cellular events have been proposed to mediate this central role of FABP4 in the regulation of metabolism in obesity and to link breast cancer risk to obesity (Hancke et al., 2010; Zeng et al., 2020).

Tumor stromal cells actively participate in breast tumor progression (Robinson et al., 2009; Kessenbrock et al., 2010). Macrophages’ function heterogeneously in the tumor stroma, exhibiting pro-tumor or antitumor effects under different circumstances (Mehla and Singh, 2019). Tumor-associated macrophages (TAMs), which play an indispensable role, are among the most abundant types of non-neoplastic cells infiltrating the TME (Condeelis and Pollard, 2006). A high density of TAMs is associated with decreased survival and poor prognosis in patients with mammary cancer. In a recent study, Hao et al. (2018a) confirmed that FABP4 is preferentially expressed in a specific macrophage subset that shows the CD11b^+^F4/80^+^MHCII^−^Ly6C^−^ phenotype. This FABP4 positive subset of TAMs directly promotes tumor growth. The intracellular expression of FABP4 in macrophages downregulates *miR-29b* via enhanced activation of NF-κB, which negatively regulates the IL-6/STAT3 signaling pathway and leads to subsequent tumor colony formation. Taken together, mammary tumor progression is enhanced by this macrophage subset, which is mechanistically accumulated in the tumor stroma through FABP4 mediated miR-29b/IL-6/STAT3 cascade.

FABP4 levels in serum are observably increased in obese subjects as a consequence of the expanded volume of adipose tissue and the subsequent upregulated release of FABP4 from adipocytes into circulation (Xu et al., 2007; Burak et al., 2015; Hotamisligil and Bernlohr, 2015). Emerging evidence suggests that elevated concentrations of FABP4 in the bloodstream are involved in breast cancer progression (Hancke et al., 2010). Circulating FABP4 initiates IL-6 expression by binding to phospholipids in the cell membrane, which serves as STAT3-activating cytokine signaling, leading to oncogenic STAT3 signaling (Yan et al., 2017). The STAT3/ALDH1 axis has recently been proven to be a new pathway for increasing the stemness of breast cancer. Circulating FABP4 functions as a tumor-promoting factor for obesity-related breast cancer through the IL-6/STAT3/ALDH1 pathway (Hao et al., 2018a).

Taken together, FABP4 represents a mechanism by which obesity promotes obesity-associated breast carcinogenesis and development through multiple bioprocesses, including supporting interactions among cancer cells, adipocytes, and tumor-associated macrophages and connecting adipokines to oncogenic signaling.

### 3.2 Ovarian cancer

A large prospective cohort study carried out by the European Prospective Investigation into Cancer and Nutrition provided solid evidence that there was an increased risk of ovarian cancer in obese women compared with normal weight women, especially among postmenopausal women (Lahmann et al., 2010). Obesity-related metabolic alterations play a critical part in the mechanisms of tumorigenesis and invasion for ovarian cancer (Ji et al., 2020). As a key modulating factor, FABP4 coordinates cellular lipid transport and response in both tumor and stromal compartments. Gharpure et al. (2018) recently revealed the potential role of FABP4 in ovarian cancer development. It has been reported that patients with high levels of FABP4 expression suffer from decreased progression-free and overall survival times compared to those with low FABP4 expression. Extensive bioinformatics analyses and a series of molecular biology experiments on cell and animal models were carried out to explore the underlying mechanisms of FABP4 on altering the tumor metastasis pattern and leading to a higher extent of invasion in ovarian cancer (Nieman et al., 2011; Gharpure et al., 2018). It was well identified that hypoxia in TME leads to a noteworthy reduction of miR-409-3p expression, which functions as a potent tumor suppressor through regulating FABP4 in ovarian cancer. The downregulation of miR-409-3p subsequently removes its inhibitory effect on FABP4, which leads to higher FABP4 levels and contributes to a poor prognosis in patients with ovarian cancer. This study provides a better understanding of the biological function of FABP4 in promoting infiltrative and invasive metastases in patients with ovarian cancer (Gharpure et al., 2018).

Furthermore, researchers utilized the small-molecule FABP4 inhibitor BMS309403 to perform a sequence of intervention studies on both cellular and animal models (Mukherjee et al., 2020). The application of BMS309403 not only alleviated the tumor burden of the syngeneic orthotopic rodent model but also increased the chemosensitivity of tumor cells to carboplatin *in vivo* and *in vitro*. These results indicate that FABP4 may be a potential therapeutic target for ovarian cancer.

### 3.3 Prostate cancer

Obesity is linked to a higher risk of advanced prostate cancer with enhanced local dissemination (Allott et al., 2013; Parker et al., 2013; Laurent et al., 2016). Increasing evidence indicates that tumor-surrounding adipocytes, also known as cancer-associated adipocytes (Dirat et al., 2011) can dramatically affect prostate cancer progression. Periprostatic adipose tissue, which has been identified as a common active endocrine organ, plays a pivotal role in the mechanism explaining the epidemiological relationship between obesity and prostate cancer progression through the secretion of adipokines. The relevance of FABP4 in prostate cancer has recently been identified. It has been proved that FABP4 treatment could promote prostate cancer cell invasion in a significant study *in vitro*, and this boosting effect was remarkably reduced by the FABP4 inhibitor. The FABP4 blocker also suppressed the subcutaneous growth of prostate cancer cells and lung metastasis in an animal model (Uehara et al., 2014). These results suggest that FABP4 can exert its effects by acting as an energy source carrier for tumor cells. FABP4 could additionally activate the phosphatidyl-inositol 3-kinase (PI3K)/AKT tumor pathway, participating in tumor growth and survival without being assimilated by prostate cancer cells (Uehara et al., 2018).

Based on these findings, the effects of FABP4 on the proliferation of metastatic prostate tumor cells were further investigated. FABP4 was highly expressed in bone metastases of prostate cancer in both obese mouse models and clinical samples. The researchers also treated metastatic prostate tumor cells with adipocyte-conditioned media and found that the expression of FABP4, IL-1β, and hemeoxygenase-1 (HMOX1) was upregulated. These findings indicated that excess adiposity could function as a fuel for metastatic neoplasm growth by transporting FAs to tumor cells through FABP4. FABP4 expression levels are increased in obesity owing to the lipid-rich TME and hyperplastic state of adipocytes, thus promoting lipid trafficking from obese adipocytes to cancer cells (Herroon et al., 2013).

However, the role of FABP4 in prostate cancer remains controversial. In contrast to peritumor-derived FABP4, mRNA *FABP4* expression is significantly decreased in tumors compared to normal prostate epithelial cell/tissue when examining human prostate cancer cell lines and clinical biopsy samples (Das et al., 2001; Tölle et al., 2011). Furthermore, overexpression of FABP4 by transfection in prostate cancer cell lines induces apoptosis by decreasing transforming growth factor-alpha activity and upregulating the expression of TNF-α, indicating a possible contribution of FABP4 in tumor suppression (De Santis et al., 2004; Hammamieh et al., 2004). Some researchers tend to believe that these differences are a consequence of the fact that the mRNA level is not completely consistent with FABP4 level in serum (Hancke et al., 2010).

### 3.4 Liver cancer

Several exposures have been confirmed as risk factors for hepatocellular carcinoma (HCC), including alcoholism, viral hepatitis, aflatoxin, and obesity (McCullough and Lloyd, 2019; Sagnelli et al., 2020). Obesity is a well-known independent risk factor of HCC. However, the mechanisms by which obesity triggers and accelerates HCC remains to be studied (Calle et al., 2003). Considering the scope of the obesity epidemic worldwide, there is a relatively looming menace to a rapid rise in obesity-related HCC (Hales et al., 2020). FABP4, which is primarily expressed in adipose tissues and macrophages, is expressed at very low levels in hepatocytes of normal diet-fed mice. However, FABP4 is robustly upregulated in an obese mouse model and obesity-promoted HCC model (Thompson et al., 2018). Analysis of the serum profile also confirmed that circulating FABP4 levels were observably improved in patients with HCC compared to healthy subjects. Furthermore, *in vitro* studies using human HCC cell lines have demonstrated that treatment with exogenous FABP4 could significantly stimulate cell proliferation. Taken together, these data indicate that liver-derived FABP4 acts as a crucial factor in the development of obesity-induced metabolic stress (hepatosteatosis) to promote hepatocarcinogenesis. A study by Attal et al. (2021) showed that the expression of FABP4 was upregulated in obesity and chronic alcohol intake, and exogenous recombinant human FABP4 (rhFABP4) stimulated proliferation and migration of HCC cells, all of which indicated the promoting role of FABP4 in HCC.

In contrast to the above findings, Zhong et al. (2018) reported that FABP4 expression was distinctly decreased in HCC tissues compared to paired normal liver tissues at both the mRNA and protein levels. It was observed that the overexpression of FABP4 significantly suppressed the process of proliferation, colony formation and even migration of the HCC cell lines *in vitro*. They also found that FABP4 restrained cancer development in a nude mouse model. Yu et al. (2000) also showed that FABP4 expression was downregulated in the cell line BEL-7404 hepatoma compared to the immortalized normal L-02 hepatic cell line. A theory has been proposed to explain the inconsistency between these reports. FABP4 may play a context-dependent role in hepatic tumor progression (McKillop et al., 2019). Upregulated hepatic or adipocyte-derived FABP4, which is recognized as exogenous FABP4, can enhance HCC progression, whereas tumor-derived endogenous FABP4 suppresses tumor progression (McKillop et al., 2019).

### 3.5 Pancreatic cancer

Pancreatic ductal adenocarcinoma (PDAC) is a rapidly progressing cancer with poor prognosis. Despite the great development of immunotherapies, targeted small molecules, and combination chemotherapy, which have vastly revolutionized tumor therapeutics, the long-term survival of patients with PDAC remains low at ∼10% without significant improvement (Siegel et al., 2020). Epidemiological research has revealed that the surrogate measure of obesity BMI is linked to the promotion of PDAC risk and poor survival (Larsson et al., 2007; Yuan et al., 2013; Chung et al., 2020). However, little information is available on the mechanisms by which obesity contributes to the occurrence and development of PDAC. Previous studies have suggested that dysregulation of FA and altered lipid metabolism are potential mechanisms (Khasawneh et al., 2009; Zaytouni et al., 2017). Luo et al. (2017) observed that FABP4 protein was significantly overexpressed in PDAC tissues compared with typical peritumoral tissues, as well as benign tumor and normal pancreatic tissues. The expression level of FABP4 was markedly higher in TNM stage III/IV PDAC cases with local invasion and lymph node metastasis than in those without lymph node metastasis, indicating that FABP4 is associated with PDAC carcinogenesis and is likely to be a predictive marker of poor prognosis. The molecular mechanisms involved should be investigated further in future studies.

### 3.6 Others

There is evidence of a strong association between obesity and colon cancer (Maruthur et al., 2012). Adipocytes are non-negligible elemental components of the TME (Bussard et al., 2016; Chen et al., 2016). Tian et al. (2020) found that FABP4 is associated with lipid accumulation in colon cancer cells, and that FABP4 overexpression promotes invasion and migration by enhancing epithelial-mesenchymal transition via the AKT pathway. However, the specific downstream signaling cascades need to be explored further. In addition, a study by Lin et al. (2020) showed that FABP4 expression level in gastric adenocarcinoma cells was significantly higher than that in normal cells. Gastric adenocarcinoma cells compete for FA uptake with tissue-resident memory T (Trm) cells in the gastric adenocarcinoma TME, and Trm cells predict a better prognosis. Few studies have focused on the function of FABP4 in lung cancer. Tang et al. (2016) found that FABP4 is expressed in non-small cell lung cancer (NSCLC) at a relatively high level and is significantly correlated with tumor TNM stage, which has prognostic value for overall survival of NSCLC. Another study showed that miR-203 could inhibit lung cancer cell metastasis by targeting FABP4 expression, which may be helpful in exploring novel targets for the diagnosis and treatment of lung cancer metastasis (Chen and Wu, 2018). In a cytological and molecular study of osteosarcoma by Zhou et al. (2020), FABP4^+^ macrophage infiltration was found in pulmonary metastatic osteosarcoma, which showed pro-inflammatory properties according to gene set variation analysis (GSVA). FABP4^+^ TAMs may be closely related to the occurrence of a pro-inflammatory TME in lung metastatic osteosarcoma cells.

Interestingly, in contrast to most obesity-promoting cancers, obesity appears to be a protective factor in melanoma, which is known as the obesity paradox. Using three public databases and comparing patients with melanoma and colon cancer, Giampietri et al. (2020) found low expression of FABP4 and other genes in patients with melanoma. This low expression may reduce the cellular internalization of fat, making patients with melanoma less sensitive to high-fat intake. In addition, loss of FABP4 expression is associated with the progression of bladder cancer. Boiteux et al. (2009) first reported that the reduced transcription of FABP4 is apparently associated with tumor clinical stage and histological grade in bladder tumors, and they confirmed for the first time that FABP4 expression is mediated by PPAR. These findings suggest that FABP4 may be a potential candidate marker for bladder cancer and that PPAR agonist-induced FABP4 expression may be a potential therapeutic strategy. A long-term follow-up study further corroborated this claim in detail (Mathis et al., 2018).

In addition, FABP4 has been linked to a number of other diseases. FABP4 is expressed in vascular endothelial cells of meningiomas, especially anaplastic meningiomas, and may serve as a potential marker (Lee et al., 2021b). A small sample size study found a negative correlation between FABP4 and cartilage thickness in end-stage knee osteoarthritis (Schadler et al., 2021). Other studies have shown that FABP4 is associated with kidney stone formation, and downregulation of FABP4 drives calcification, suggesting a link between kidney stones and metabolic syndrome (Taguchi et al., 2020). Conversely, FABP4 promotes renal interstitial fibrosis through inflammation and lipid metabolism, and is also a potential drug target (Qiao et al., 2019). It is worth noting that in obstetrics and gynecology, FABP4 also plays a biological role in the pregnancy process, down-regulation of FABP4 may promote abortion, and FABP4 can regulate the uterine volume of sexually affected pregnancy (Wang et al., 2017).

## 4 Conclusion

Obesity and subsequent dysfunction of adipose tissue have been associated with an increased risk of various malignancies. The altered levels of adipokines provide a potential mechanism for the clear epidemiological association between obesity and cancer. In this respect, many studies have attempted to demonstrate the role of adipokines in tumorigenesis and development. FABP4, also known as A-EABP, which is expressed predominantly in adipose tissue and macrophages, has become the most studied FABP family member because of its notable function in obesity-related diseases. However, the specific relationship between FABP4 and various cancers is still unclear, and the specific mechanism of FABP4 on cancer still needs to be explored. Although it has been well documented that FABP4 plays a pivotal role in the tumorigenesis and invasion by interacting with multiple classic pathways, such as PI3K/Akt and STAT3/ALDH1 signaling. In addition, it is known that in immune cells, FABP5 is expressed in a variety of immune cells, while FABP4 is almost exclusively expressed in macrophages. In previous studies, it was found that FABP4 could promote cholesterol accumulation in macrophages. And as in adipocytes, FABP4 increases the production of inflammatory cytokines in macrophage. It has shown that FABP4 is preferentially expressed in specific subtypes of macrophages. This FABP4 positive subset of TAMs directly promote tumor growth (Hao et al., 2018a). A recent study found that FABP4 could promote macrophage death by ceramide production, contributing to the chronic inflammation in obesity, which may also be a potential cancer-promoting mechanism (Zhang et al., 2017). Another study has found that high levels of FABP4 predict a poor prognosis for neuroblastoma. Knockdown of FABP4 regulates the differentiation of macrophages and inhibit tumor, suggesting that FABP4 could enhance tumor progression through macrophage differentiation (Miao et al., 2021). It is worth noting that despite the intracellular FABP4 being initially discovered, circulating FABP4 secreted by adipocytes and macrophages also functions as a novel adipokine by activating different oncogenic pathways.

Although there has been accumulating evidence supporting a potential role of FABP4, produced by fat tissues and/or macrophages, in mediating obesity-related cancer development and progression, further studies are needed to elucidate the detailed molecular mechanisms by which cytoplasmic and circulating FABP4 expression interfere with different signaling pathways. While most studies have reported that elevated FABP4 levels are associated with increased tumor aggressiveness, other studies have observed that loss of FABP4 is related to increased progression and worse outcomes in some cancers. One possible explanation for this discrepancy may be that the overexpression or knockdown of FABP4 using molecular biological techniques in cell models is factitious, whereas aberrant FABP4 expression in the obese state or disease metastasis is induced rather than forced. Therefore, the involvement of FABP4 was not “one-direction” or monotonous when trying to unravel the role of FABP4 in obesity-related carcinogenesis in either *in vitro* or *in vivo* studies. So far, FABP4 inhibitors have only been used *in vitro* and animal studies, and their risk and efficacy on human remain unknown. There is still a lot of work to be done to translate FABP4 products into clinical applications. FABP4 actively participates in dysregulated metabolism in patients with cancer through complex multisystem interactions with various signaling pathways. A deeper and more comprehensive understanding of how FABP4 signaling is involved in obesity-associated tumors could improve the potential of developing new therapeutic targets and strategies for tumor treatment. The molecular mechanisms, the feasibility of biomarkers and the application of drugs with relevant targets warrants further investigation in follow-up studies on the basis of these current findings.
